# Keeping an eye on Ca^2+^ signalling to tackle dry eye diseases

**DOI:** 10.1016/j.ebiom.2021.103741

**Published:** 2021-12-10

**Authors:** Flore Sneyers, Jens Loncke, Geert Bultynck

**Affiliations:** KU Leuven and Leuven Kanker Institute, Laboratory of Molecular and Cellular Signaling, Department of Cellular and Molecular Medicine, Campus Gasthuisberg O/N-I bus 802, Herestraat 49, BE, 3000 Leuven, Belgium

**Keywords:** CISD2, CDGSH Iron Sulfur Domain 2, CLSC, corneal limbal stem cell, CsA, cyclosporin A, ER, endoplasmic reticulum, IP3R, IP3 receptor, NF-AT, Nuclear Factor of Activated T cells, ROS, reactive oxygen species, RyR, ryanodine receptor, SERCA, sarco/endoplasmic reticulum Ca^2+^ ATPase

An early consequence of ageing in humans is a decline in vision. Usually, this is limited to presbyopia starting at the age of forty. However, at later age, more severe age-related eye disease can occur, possibly resulting in blindness. Proper corneal epithelium maintenance is indispensable for corneal clarity and good vision quality. A major culprit of blindness associated with corneal epithelium defects is the formation of corneal scars. These scars are the outcome of a wound healing process initiated by re-epithelialisation and further driven by activated keratocytes, which help to re-establish the integrity of the tissue but fail to restore optical properties.

A protein key to healthy ageing in mammalian organisms is CDGSH Iron Sulfur Domain 2 (CISD2) [1]. CISD2-protein levels are dynamically regulated whereby adequate levels of CISD2 prevent early ageing and prolongs lifespan. Moreover, loss-of-function mutations in the CISD2 gene cause Wolfram syndrome 2, a rare genetic disease [2]. From a biochemical perspective, CISD2 is an iron-sulfur cluster-binding protein affecting reactive oxygen species (ROS) formation. From a cell physiological perspective, CISD2 is strategically located at the endoplasmic reticulum (ER) and mitochondria interface, key organelles for protein folding, Ca^2+^ signalling, bioenergetics and cell survival. Thus, given the crucial role of ER-mitochondrial Ca^2+^ signalling in health and disease, CISD2 is uniquely positioned to impact cellular fitness. However, the exact mechanisms by which CISD2 influences intracellular Ca^2+^-transport systems remain unclear.

Inspired by their previous findings in CISD2-deficient mice suffering from blindness due to corneal damage [3] and resembling clinical manifestations associated with corneal limbal stem cell (CLSC) deficiency, Tsai et al. studied CISD2 levels in the corneal tissues taken from patients undergoing corneal transplantation procedures [4] (see Fig. 1). Notably, CISD2 was decreased in the corneal epithelium derived from patients with corneal epithelium deficits but not from those with corneal defects stemming from endothelial problems. *Vice versa*, CISD2-deficient mice displayed corneal epithelium deficits due to a vicious cycle of inflammation and continuous loss of epithelial cells, further aggravated by impaired renewal and differentiation of epithelial cells from CLSC. Comparing young *versus* old mice, the authors revealed a continuous proliferation of CLSCs compensating for the increased loss of epithelial cells in young CISD2-knockout mice, eventually resulting in CLSC depletion and accumulation of corneal epithelium damage. Next, the authors revealed that corneal epithelial cells lacking CISD2 displayed higher basal cytosolic Ca^2+^ levels, resulting in augmented calcineurin activity and increased expression of genes driven by nuclear factor of activated T-cells (NF-AT), a transcription factor activated by calcineurin. Together with reduced mitochondrial metabolism and increased ROS production, loss of CISD2 impaired epithelial cell migration and wound healing. Interestingly, cyclosporin A (CsA) and FK506, two calcineurin inhibitors of independent classes, rescued these features and normalised basal cytosolic Ca^2+^ levels, thereby suppressing NF-AT signalling. Not only did CsA restore corneal functionality in CISD2-deficient mouse models, it improved clinical outcomes of patients with corneal defects due to severe dry eye disease. Here, the authors enrolled five patients with dry eye disease, who were topically treated with a more concentrated CsA solution compared to the standard (0.1% instead of 0.05%). Clinical analysis revealed improved corneal epithelium integrity and functionality, reducing dry eye characteristics. Hence, this study advocates revisiting the clinical treatment regimens of patients with dry eyes by augmenting the CsA concentration in topic applications. Although a modest increase from 0.05% to 0.1% already had a beneficial outcome on the corneal functionality, CsA concentrations up to 2% are considered safe for such treatments [5]. This work further underpins the importance for the tight regulation of Ca^2+^ signalling in human health, as also reduced Ca^2+^ signalling have been implicated in dry eye disease. Indeed, mice lacking IP_3_ receptors isoforms 2 (IP_3_R2) and 3 (IP_3_R3) display dry eye disease [6], while cells isolated from salivary glands from Sjögren's syndrome patients, who also suffer from dry eyes symptoms, display a reduction in IP_3_R2 and IP_3_R3-protein levels and a concomitant decrease in IP_3_R-mediated Ca^2+^ release [7].

**Fig. 1:**
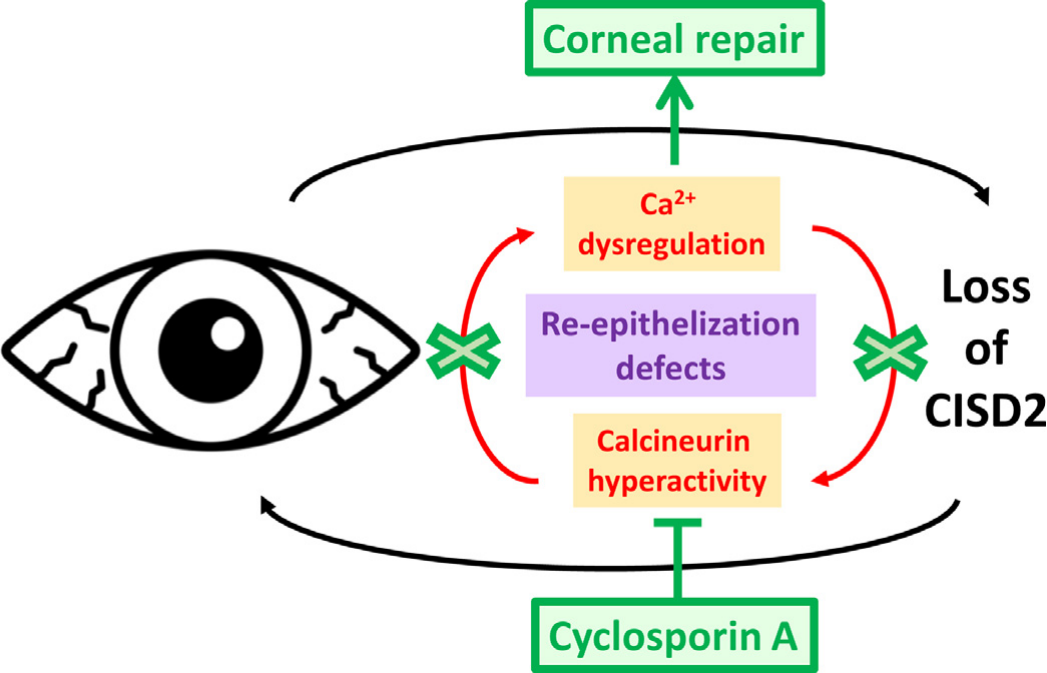
Loss of CISD2 appears to be an important aspect in the pathogenesis of corneal epithelial diseases that result in severe dry eye symptoms. The deleterious effects of the decline in CISD2-protein levels are fueled by a vicious feed forward cycle (red arrows) driving dysregulation of intracellular Ca^2+^ homeostasis & dynamics and calcineurin hyperactivity. Topical application of clinically available calcineurin-inhibiting drugs such as the immunosuppressant cyclosporin A holds therapeutic promise to halt or at least impede this cycle (green arrows/crosses), thereby promoting corneal repair and improving clinical features of dry eye disease. [The picture of the eye was taken from the Noun project, i.e. an inflamed eye by Llisole from the Noun Project; thenounproject.com].

Many questions remain, representing exciting future research avenues.

It remains unclear how CISD2 sustains the function of intracellular Ca^2+^-transport systems and thus enables adequate intracellular Ca^2+^ homeostasis and dynamics in corneal epithelial cells. These additional CISD2 targets may provide additional strategies to normalise Ca^2+^ signalling in the cornea, possibly including pumps such as Ca^2+^ sarco/endoplasmic reticulum Ca^2+^ ATPases (SERCAs) and intracellular Ca^2+^-release channels such as IP_3_Rs and ryanodine receptors (RyRs) (reviewed in [2]). For instance, in CISD2-deficient neuronal cell models, dantrolene, an FDA-approved RyR inhibitor, prevented cell damage caused by leaky RyR channels [8]. Moreover, it is fascinating that CsA is able to normalise Ca^2+^ dynamics. This further supports the concept that, beyond transcriptional programs, hyperactive calcineurin can directly modulate the properties of Ca^2+^-transport systems.

The underlying mechanisms downregulating CISD2-protein levels remain to be established. Additionally, it is not clear whether only CISD2 proteins or also other proteins are declined in dry eye diseases. Such insights may provide novel opportunities to elevate CISD2 levels, thereby boosting the repair of corneal epithelium defects. This might be feasible, as studies focusing on spinal cord injuries demonstrated that curcumin could counteract injury-induced downregulation of CISD2 and subsequent inflammation [9].

Finally, Wolfram syndrome type 1 and 2 are caused by mutations in WFS1 and CISD2, respectively, and are both characterised by optical atrophy, diabetes type 1 and deafness. It is also clear that their converging role in Ca^2+^ signalling dysregulation might underlie commonalities in the pathogenesis and disease outcomes upon loss of function of WFS1 or CISD2 [2]. To date, corneal abnormalities in Wolfram syndrome have only been reported in patients carrying WFS1 mutations [10]. Therefore, it could be interesting to examine whether also WFS1 is affected in dry eye diseases and whether WFS1 could contribute to corneal epithelium regeneration.

## Contributors

GB conceived this invited commentary with further input from FS and JL. All authors contributed to the writing.

## Declaration of Competing Interest

None of the authors have any competing interests to declare.
